# Predictors of the inability to achieve full oral feeding in postoperative infants with CHD

**DOI:** 10.1017/S104795112300313X

**Published:** 2023-08-23

**Authors:** Marin Jacobwitz, Sharon Y. Irving, Helene Moriarty, Jennifer Yost, Arastoo Vossough, Daniel J. Licht, Jennifer M. Lynch

**Affiliations:** 1 Division of Neurology, Children’s Hospital of Philadelphia, Philadelphia, PA, USA; 2 M. Louise Fitzpatrick College of Nursing, Villanova University, Villanova, PA, USA; 3 Critical Care Nursing, Children’s Hospital of Philadelphia, Philadelphia, PA, USA; 4 Department of Family and Community Health, University of Pennsylvania School of Nursing, Philadelphia, PA, USA; 5 Perelman School of Medicine at the University of Pennsylvania, Philadelphia, PA, USA; 6 Division of Neuroradiology, Children’s Hospital of Philadelphia, Philadelphia, PA, USA; 7 Division of Cardiothoracic Anesthesiology, Children’s Hospital of Philadelphia, Philadelphia, PA, USA

**Keywords:** CHD, oral feeding dysfunction, neonatal cardiac surgery, neonatal

## Abstract

**Objectives:**

Poor oral feeding is a known contributor to growth challenges in neonates with complex CHD who require early surgery. Almost 60% of these infants do not achieve full oral feeding by hospital discharge. This study’s objective was to identify predictors of the inability to achieve full oral feeding by discharge in neonates with complex CHD following surgical intervention with cardiopulmonary bypass.

**Study Design:**

A retrospective analysis of a prospective study of 192 full-term neonates with complex CHD was performed. A stepwise selection logistic regression model was developed to predict oral feeding status at hospital discharge. Univariate subgroup analysis was performed with groups determined based on a CHD classification system.

**Results:**

58% of neonates (112/192) failed to achieve full oral feeding by hospital discharge. A logistic regression model identified duration of deep hypothermic circulatory arrest and reintubation as predictors of the inability to achieve full oral feeding. Among neonates who achieved full oral feeding by discharge (42%), only 7.5% did so after postoperative day 10. Brain maturation, brain injury, and preoperative oral feeding were not predictors of full postoperative oral feeding.

**Conclusions:**

Many infants with CHD fail to achieve full oral feeding by time of hospital discharge. Longer duration of deep hypothermic circulatory arrest and increased number of intubations were predictive of poor feeding after surgery. Prolonging hospitalisation solely to achieve full oral feeding after postoperative day ten is of limited utility; earlier discharge should be promoted to avoid negative impacts on neonatal neurodevelopment as unintended consequences of lengthy hospitalisations.

CHD is the most common congenital defect, affecting approximately nine per 1000 live births, with nearly three per 1000 infants classified as having critical CHD, requiring surgical intervention in the neonatal period.^1,2^ Growth failure and malnutrition are common consequences of CHD, with up to 70% of neonates with CHD characterised as malnourished.^3^ In children without CHD, growth failure during early infancy has been identified as a risk factor for impaired neurodevelopment, specifically in poor school performance, executive function deficits, and developmental delay.^4–7^ The risk for growth failure in infants and children with CHD is likely multifactorial, often attributed to such factors as gastroesophageal reflux, malabsorption, swallowing dysfunction, hypermetabolic state, inadequate caloric intake, genetic syndrome, and/or immaturity of the gastrointestinal system.^8^ Empirical evidence reveals that normal preoperative oral feeding does not predict successful postoperative oral feeding,^8^ suggesting there are further vulnerabilities acquired by neonates with complex CHD from birth through surgery to the postoperative period.^9^ The cause of the decline in their oral feeding abilities is unclear and is an area ripe for further evaluation.^9^

There are several preoperative, intraoperative, and postoperative risk factors that may explain poor oral feeding in neonates with complex CHD who have undergone surgery. Previously reported examples of these risk factors include the presence of genetic syndromes, structurally immature brains at birth, and acquired brain injury.^10^ A recent scoping review of the literature identified that across 25 studies only a limited number of risk factors and health-related outcomes (n = 78) were evaluated for their association with poor postoperative oral feeding in infants and neonates with CHD.^11^ None of the pre-, intra-, or postoperative risk factors were consistently associated with poor oral feeding. It is noteworthy that only one study in this review evaluated brain MRI data and its association with oral feeding outcomes in neonates with hypoplastic left heart syndrome.^11,12^ This study found that slower postnatal brain maturation, but not perioperative brain injury, was associated with oral feeding difficulties.^13^ The only health-related outcomes consistently associated with poor oral feeding were length of hospital stay (9/10 studies, or 90%) and length of ICU stay (3/4 studies, or 40%).^11^ The aim of the present study was to identify the predictors of poor postoperative oral feeding in neonates with complex CHD who require surgical intervention for survival examining prenatal, preoperative, intraoperative, and postoperative factors. A sub-aim was to evaluate the postoperative oral feeding trajectory of the subjects based on cardiac lesion type and if this trajectory varied within the cohort.

## Materials and methods

### Design and study population

This study is a retrospective analysis of a prospective study that evaluated risk factors for development of brain white matter injury in 192 full-term neonates with complex CHD who have undergone surgical intervention requiring cardiopulmonary bypass in the neonatal period. The study cohort was recruited and enrolled between 2008 and 2018. Enrollment was open to any parent with a fetus or neonate with CHD with a plan for surgical intervention with cardiopulmonary bypass within the neonatal period. Throughout the enrollment period, surgical strategy remained consistent within the institution, with deep hypothermic circulatory arrest as the only surgical perfusion strategy utilised for arch reconstruction. Inclusion criteria were full-term neonates (≥ 36 weeks gestation) with complex CHD who were medically stable for at least 24 hours prior to enrollment for the study. Exclusion criteria reflect potential clinical confounders known to impact brain development and/or pose additional risk factors for brain injury. Exclusion criteria were as follows: neonatal depression (five-minute APGAR < 5 or umbilical cord arterial blood gas pH < 7.0); small for gestational age (< 2 kg birth weight); perinatal seizures; evidence of end-organ injury (liver function tests > 2 times normal for age; creatinine > 2 mg/dL; heart failure); preoperative cardiac arrest requiring cardiopulmonary resuscitation; and grade III or IV intraventricular haemorrhage. Inclusion and exclusion criteria served to eliminate those subjects that would be considered sicker than the typical neonate with CHD. Additionally, those infants with an incomplete dataset were excluded from this retrospective analysis.

To enable subgroup analysis, subjects were categorised based on cardiac lesion type according to the CHD grading system.^14^ This grading system, referred to as primary CHD grade, includes grade I (two ventricles, normal aortic arch), grade II (two ventricles, arch obstruction), grade III (single ventricle, normal arch), and grade IV (single ventricle, arch obstruction).^14^ For the purposes of this study, residual cardiac lesion was defined as any unintended residual intracardiac shunts identified on postoperative echocardiography.

### Data collection

Institutional Review Board approval was obtained. All data were entered into a Research Electronic Data Capture (REDCap) database (Vanderbilt University, 2004). Data were collected via chart review from paper charts for all subjects enrolled prior to 2011 or from electronic health records for all subjects enrolled after 2011. All preoperative and postoperative brain MRI images for the primary study were deidentified and reviewed by two independent reviewers (DJL, AV) to ensure consistency in brain MRI interpretation across subjects. Methods for white matter injury and whole brain volume segmentations are available in Supplemental Materials: MRI Methods. For this retrospective analysis study, independent variables for consideration were selected by applying knowledge of the existing evidence^11^ and agreement of clinical experts (see Supplementary Table 2).

### Statistical analysis

The dependent variable was a dichotomous variable defined by whether or not the patient achieved full oral feeding at hospital discharge. Failure to achieve full oral feeding was defined as the need for any supplemental enteral tube nutrition upon hospital discharge (i.e., nasogastric or surgical gastrostomy tube). A stepwise variable selection was performed to determine a multivariate logistic regression model to predict inability to achieve oral feeding. In 2016, a feeding pathway was instituted in which nasogastric tubes were inserted postoperatively to promote enteral nutrient intake, and support weight gain for the neonates during recovery post cardiopulmonary bypass. Given this change in practice, an analysis was completed to determine if there was an influence on oral feeding outcomes in the study cohort. A Wilcoxon rank-sum test was used to compare all continuous variables between subjects who did and did not achieve oral feeding prior to discharge. Statistical significance was set at a *p* value of 0.003 after performing the Bonferroni correction. Statistical analyses were performed with MATLAB (2020b, MathWorks). A sample size calculation was performed to ensure that statistical analyses were adequately powered.

## Results

### Sample description

In the study cohort, 58% of subjects (112/192) were male with a median birth weight of 3.34 kg [IQR 3–3.67 kg] and a median birth head circumference of 34 cm [IQR 33–35 cm]. Fifty-eight percent of subjects were born via vaginal delivery, with pregnancy complications affecting 26% (49/192) of mothers (see Table 1). A prenatal diagnosis of CHD occurred in 88% (169/192) of subjects, with the most common being hypoplastic left heart syndrome in 37% (70/192) and d-transposition of the great arteries in 33% of subjects (64/192; see Table 1). A confirmed chromosomal disorder was identified in 6% and suspected in 20% (38/192) of subjects. Primary CHD grades were as follows: Grade I: 74/192 (39%), Grade II: 33/192 (17%), Grade III: 4/192 (2%), and Grade IV: 81/192 (42%). Preoperative cardiac catheterisation was required in 22% (42/192), and 20% (38/192) required non-procedural, preoperative intubation (see Table 1). See Table 1 for details on intraoperative and postoperative descriptive variables of the cohort.

Of the 192 subjects studied, 138 (72%) had preoperative oral intake, with only 3 subjects (2%) achieving full oral feeding preoperatively. Out of the whole cohort, 112 subjects (58%) did not achieve full oral feeding by time of hospital discharge, 73 of whom (65%) had preoperative oral intake. Based on primary CHD grade, those that did not achieve full oral feeding by time of hospital discharge was as follows: Grade I 23/74 (31%); Grade II 27/33 (82%); Grade III 1/4 (25%); and Grade IV 61/81 (75%). There was no statistical difference in the incidence of inability to achieve oral feeding following the institution of a feeding pathway to promote improved nutrition through the use of nasogastric tubes in the immediate postoperative period (Pearson’s chi square = 0.007; p value = 0.93).

### Univariate analysis

**Figure 1.**
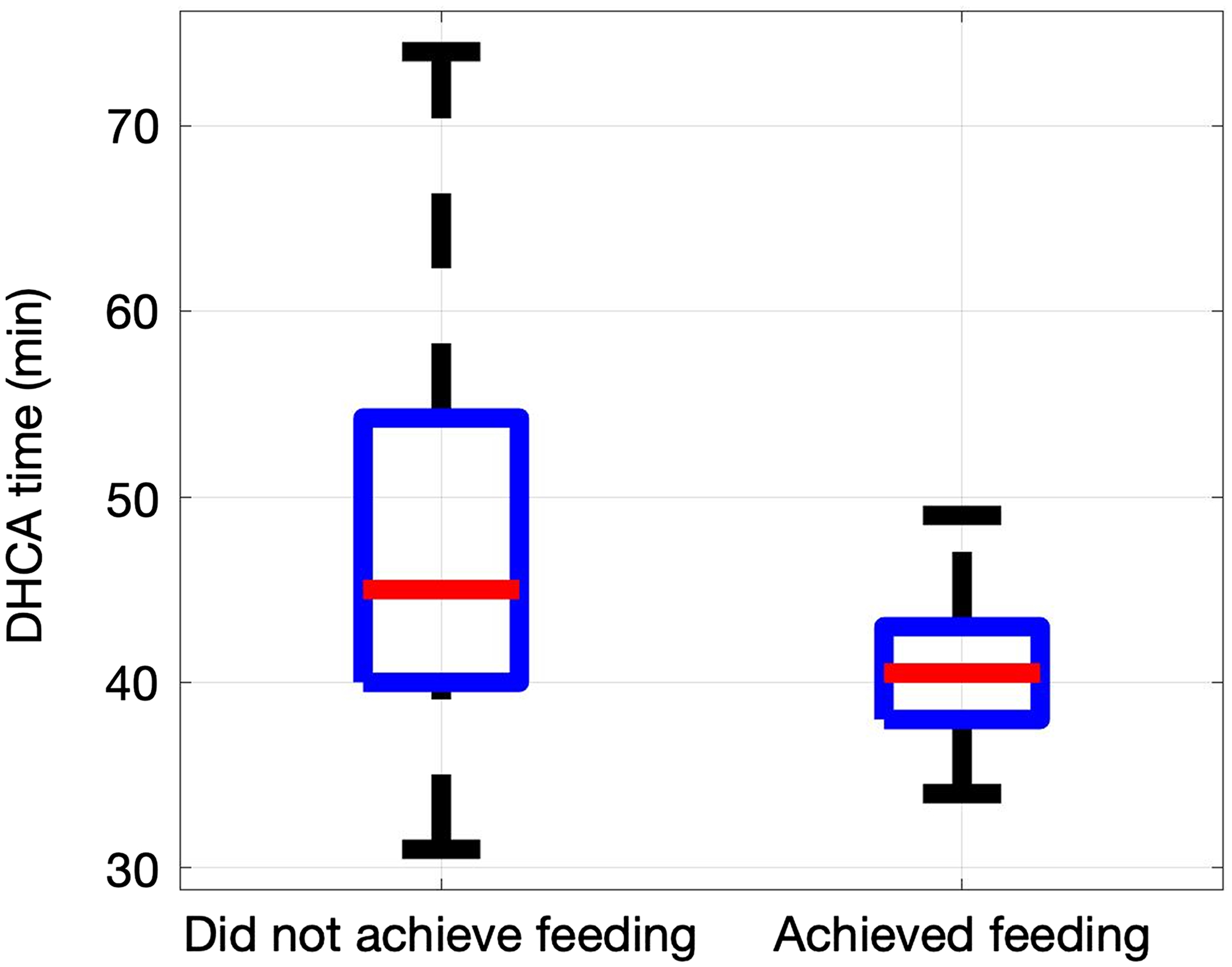
Box and whisker plot demonstrating deep hypothermic circulatory arrest (DHCA) as a predictor of oral feeding ability for CHD grade IV based on subgroup univariate analysis.

In a univariate analysis, 19 variables were statistically significantly different between neonates that did achieve full oral feeding prior to hospital discharge and those who did not. Subgroup analysis of the cohort was then performed with primary CHD grade (excluding primary CHD grade III given the small sample in this group, n = 4 subjects). Table 2 identifies the variables significantly associated with CHD grade and inability to achieve oral feeding, including p values. See Supplementary Table 1 for p values of all variables in the univariate analysis. Of note, none of the MRI results of structural (total maturation score, brain volumes, quartered point system score)^10,15^ and acquired (white matter injury, arterial ischaemic stroke, intracerebral haemorrhages) brain abnormalities were significantly correlated with failure to achieve oral feeding prior to discharge. Of the neonates requiring deep hypothermic circulatory arrest (n = 117), the median duration for CHD grade II was 32 minutes (IQR 25.75–39) and 44 minutes (IQR 39–51) for CHD grade IV (p < 0.001). Longer duration of deep hypothermic circulatory was associated with worse postoperative oral feeding outcomes for CHD grade IV (p = 0.0016, Fig 1).

### Multivariate analysis

Stepwise selection of a multivariate logistic model yielded two independent variables that were significantly associated with failure to achieve full oral feeding: the duration of deep hypothermic circulatory arrest (deep hypothermic circulatory; p value<0.0001) and number of endotracheal intubations (p value 0.0147) as described by the formula (AUC 0.8):

Log odds of Achieving Full Oral Feeding Before Discharge = 1.98–0.0564x(minutes of DHCA)–0.7125x(number of intubations)

Of the neonates in the entire cohort who required more than one intubation, 76% (28/37) did not achieve full oral feeding by hospital discharge, compared to 54% (84/155) of the neonates who required only one intubation. Importantly, vocal cord paralysis was not predictive of worse feeding outcomes. Deep hypothermic circulatory duration, however, is only applicable to a subgroup of subjects who required deep hypothermic circulatory for aortic arch reconstruction (see subgroup analysis above).

### Time to oral feeding

**Figure 2.**
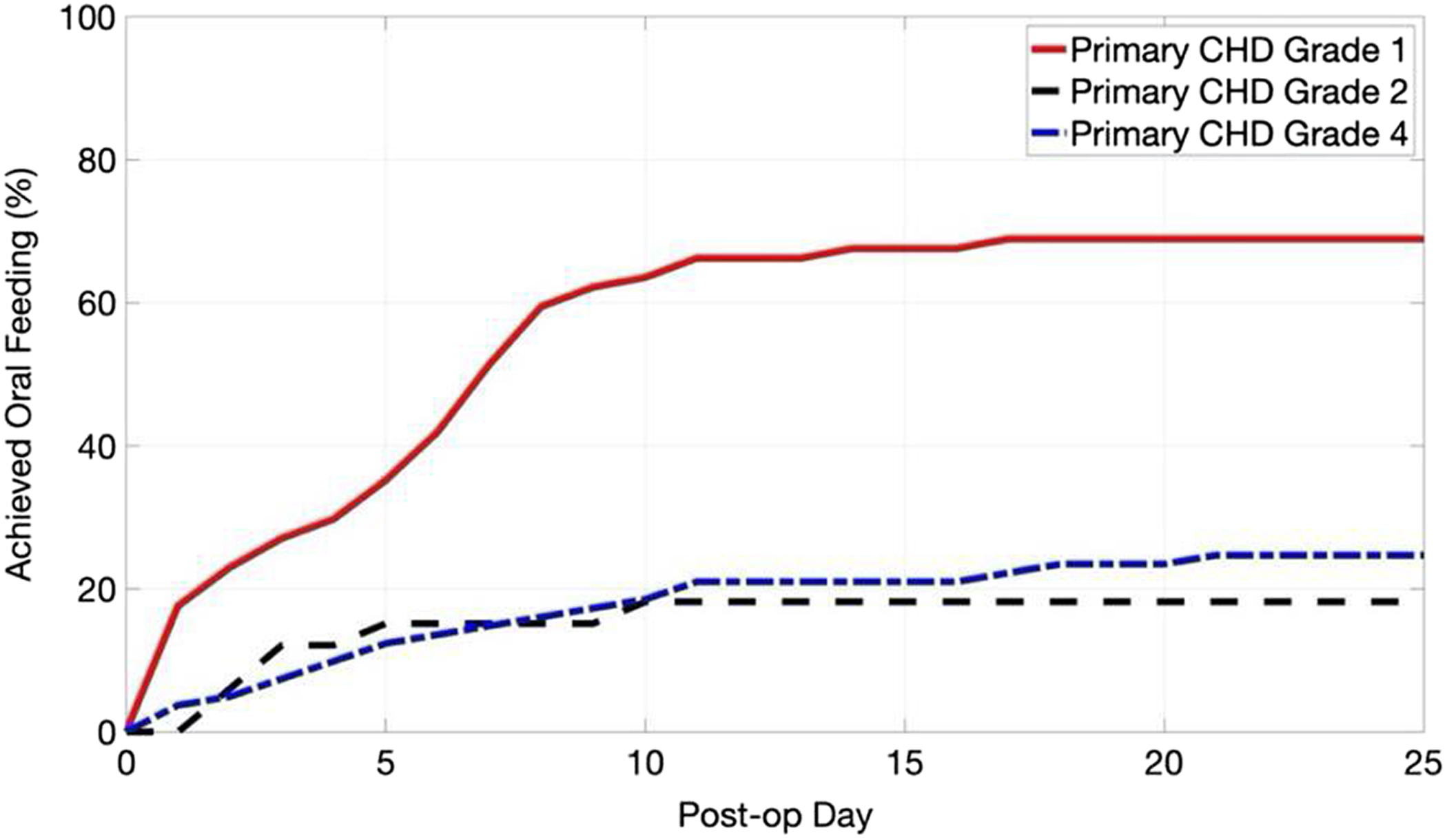
Kaplan–Meier survival curve demonstrating that, among neonates who achieved full oral feeding by discharge (42%), only 7.5% did so after postoperative day 10.

Of the 42% of subjects who did achieve oral feeding by discharge, 89% achieved full oral feeding prior to postoperative day 10 (see Fig 2). Only 7.5% of the cohort achieved full oral feeding thereafter, regardless of cardiac lesion. Based on CHD grade, if full oral feeding was not achieved by postoperative day 10, the percentage of subjects who then achieved full oral feeding by hospital discharge, was as follows: 14.8% for CHD grade I; 0% for CHD grade II; and 7.6% CHD grade IV.

## Discussion

This study utilised a comprehensive dataset of prenatal, preoperative, intraoperative, and postoperative factors to allow for a novel exploration of predictors of the inability to achieve full oral feeding by time of hospital discharge in neonates with critical CHD who required surgical intervention with cardiopulmonary bypass (see Supplementary Table 2). Findings revealed that longer duration of deep hypothermic circulatory and reintubation were predictive of the inability to achieve full oral feeding at time of discharge in neonates with complex CHD. If full oral feeding was not achieved by postoperative day ten, the likelihood of achieving full oral feeding by time of hospital discharge declined substantially regardless of cardiac lesion type. Preoperative oral feeding did not predict postoperative oral feeding ability consistent with published literature.^8^

Duration of deep hypothermic circulatory was a strong predictor of failure to feed for CHD grade IV, but not CHD grade II, likely due to shorter duration of DHCA in neonates with primary CHD grade II. The median duration of deep hypothermic circulatory for neonates requiring deep hypothermic circulatory who achieved full oral feeding was 41 minutes compared to 47 minutes for those who did not. This finding is consistent with the literature on neurodevelopmental outcomes and duration of deep hypothermic circulatory.^16^ Wypij et al. (2003) evaluated the association between length of deep hypothermic circulatory and risk of neurodevelopmental dysfunction using the cohort of children with transposition of the great arteries (d-TGA) from the Boston Circulatory Arrest Trial.^16^ Developmental, speech, and neurologic outcomes were evaluated at eight years of age utilising a variety of validated child development testing tools.^16^ Deep hypothermic circulatory was not associated with negative neurodevelopmental outcomes until circulatory arrest reached the threshold of 41 minutes, with outcomes steadily worsening as deep hypothermic circulatory time exceeded 41 minutes.^16,17^ Identifying deep hypothermic circulatory duration as a predictor for poor postoperative oral feeding is important knowledge for the cardiac community. As a modifiable factor, this finding supports the need for continued investigation into the impact of surgical strategies on neurodevelopmental outcomes.

Reintubation during the neonatal hospitalisation was also predictive of poor oral feeding outcomes, although the exact mechanism behind this association requires further investigation. Of note, vocal cord paralysis was not predictive of successful postoperative oral feeding in this study. A previous study found that neonates with a nasotracheal tube for cardiac surgery had better oral feeding outcomes when compared with neonates with oral endotracheal tubes.^18^ The number of endotracheal intubations has not been considered in prior studies based on a recent scoping review evaluating oral feeding dysfunction in neonates post cardiac surgery.^11^ However, four studies did evaluate if failed postoperative extubation, which is likely a surrogate for number of intubations, impacted oral feeding dysfunction.^11,19–22^ Only one of the four studies found a significant association between failed postoperative extubation and oral feeding dysfunction specifically in neonates with single ventricle physiology.^22^

Interestingly, brain MRI characteristics were not predictive of oral feeding outcomes in this cohort of neonates with complex CHD post cardiac surgery. Although it is known that the brains of full-term neonates with CHD are structurally immature,^10^ preoperative total maturation score, presence of white matter injury, total brain volume, and quartered point score (a white matter injury injury severity score^15^) were not predictive of oral feeding ability at the time of discharge. This may be due to the notion that supratentorial brain injury as a result of complex CHD may be less directly related to swallowing functional control, which occurs in the lower brainstem.^23^

Perhaps the most striking finding of this study is that, regardless of heart anatomy or surgical strategy, the majority of neonates who did achieve oral feeding by discharge did so by postoperative day 10 (see Fig 2). These data suggest that prolonging hospitalisation for the goal of improving oral feeding skills despite medical readiness for discharge may not be the optimal approach for this population of neonates. Given that there is a paucity of literature that has previously evaluated the oral feeding trajectory of neonates with CHD within the immediate postoperative period utilising similar statistical techniques, this is a novel addition to the literature. Earlier discharge home may foster improved neurodevelopment; therefore, emphasis should be placed on education for families and discharge planning early postoperatively.^24,25^ Neurodevelopmental skills excel once in a stimulating home environment and, despite best efforts, this cannot be replicated in a hospital environment.^26–28^ Therefore, practice needs to shift to providing education to families about nasogastric tubes and tube feeding upon diagnosis of CHD, continuing this education through to the postoperative period, and prioritising discharge home with alternative feeding support (e.g. nasogastric tube) if medically stable and the neonate has not achieved oral feeding by postoperative day ten regardless of cardiac lesion.

This study has several limitations to consider. First, this study was based on a cohort from a single institution, which may impact the generalisability to populations at other institutions. Second, because this is a retrospective analysis of a prospective study, there are variables that were not included in the dataset that may be predictive of the inability to achieve full oral feeding, such as infant weight gain and nutrient intake. Third, although identification of the percentage of subjects who had delayed discharge primarily for oral feeding difficulties would have contributed to this manuscript, it was not possible to attain this data retrospectively in an accurate manner given medical complexity of the population. Lastly, there is no standardised oral feeding protocol to improve postoperative oral feeding at the study institution; therefore, the variation in practice of individual clinicians could be considered a limitation. However, this was unable to be quantified. Despite the acknowledged limitations, the large cohort and breadth of variables in the study dataset are more comprehensive relative to other similar studies.^11^

## Conclusion

Longer duration of deep hypothermic circulatory and reintubation are predictive of the inability to achieve full oral feeding by the time of hospital discharge in neonates with complex CHD post cardiac surgery. Regardless of cardiac lesion type, the majority of infants who achieve full oral feeding by hospital discharge do so prior to postoperative day 10. Therefore, prolonging hospitalisation to improve the oral feeding ability is of limited utility after postoperative day 10 and earlier discharge to home should be encouraged to promote positive neurodevelopment in the home environment.

## Supplementary Material

Supplementary 1

Supplementary 2

The supplementary material for this article can be found at https://doi.org/10.1017/S104795112300313X.

## Figures and Tables

**Table 1.** Preoperative, intraoperative, postoperative characteristics.

Variable	N (%) Median [IQR]
Gender	
Female	80 (42%)
Male	112 (58%)
Race	
African American/Caribbean/African	21 (11%)
Asian	4 (2%)
Caucasian	145 (76%)
Mixed	3 (2%)
Other	17 (9%)
Ethnicity	
Latino	14 (7%)
Other	178 (93%)
Type of Birth	
Vaginal	112 (58%)
C-Section	80 (42%)
Pregnancy Complications	
Gestational Diabetes	12 (6%)
Gestational Hypertension	11 (6%)
Pre-Eclampsia	2 (1%)
Other*	31 (16%)
None	143 (75%)
Birth Weight (kg)	3.34 [3-3.67]
Birth Head Circumference (cm)	34 [33-35]
Time of Cardiac Diagnosis	
Prenatal	169 (88%)
Postnatal	23 (12%)
Primary Diagnosis	
Hypoplastic Left Heart Syndrome	70 (37%)
d-Transposition of the Great Arteries	64 (33%)
Interrupted Aortic Arch	8 (4%)
Truncus Arteriosus	2 (1%)
Tetralogy of Fallot with Pulmonary Atresia	8 (4%)
Coarctation with Ventricular Septal Defect	2 (1%)
Ebstein’s Anomaly	1 (1%)
Unbalanced Atrio Ventricular Canal	7 (4%)
Double Inlet Left Ventricle	3 (2%)
Double Outlet Right Ventricle	7 (4%)
Aortic Arch Hypoplasia with Ventricular Septal Defect	8 (4%)
Aortic Arch Hypoplasia with Coarctation	8 (4%)
Tetralogy of Fallot without Pulmonary Atresia	1 (1%)
Tricuspid Atresia	2 (1%)
Primary CHD Grade	
Grade I (two ventricles, normal arch)	74 (39%)
Grade II (two ventricles, arch obstruction)	33 (17%)
Grade III (one ventricle, normal arch)	4 (2%)
Grade IV (one ventricle, arch obstruction)	81 (42%)
Preoperative Cardiac Catheterisation	
Yes	42 (22%)
Balloon	37/42 (88%)
Stent	4/42 (10%)
Other	1/42 (2%)
Preoperative Non-Procedural Intubation	
Yes	38 (60%)
No	25 (40%)
Chromosomal Disorder	
Yes	11 (6%)
No	143 (75%)
Suspected	38 (20%)
Extra-Cardiac Anomalies	
Yes	66 (34%)
No	126 (66%)
Age at Surgery (days)	4 [3–5]
Total Support Time (Cardiopulmonary Bypass þ	81 [69–99]
DHCA; minutes)	
Duration of Cardiopulmonary Bypass (minutes)	41 [38–49]
Additional Cardiopulmonary Bypass	
Yes	17 (9%)
Duration of Aortic Cross Clamp (minutes)	44 [38–59]
Additional Aortic Cross Clamp	
Yes	4 (2%)
DHCA	
Yes	117 (61%)
No	75 (39%)
Duration of DHCA (minutes)	
Primary CHD Grade II	32 [25.75–39]
Primary CHD Grade IV	44 [39–51]
Additional DHCA	
Yes	2 (2%)
Duration of Cooling (minutes)	16 [15–20]
Duration of Rewarming (minutes)	23 [22–27]
Lowest Temperature (Celsius)	18.7 [18–28.3]
Type of Cardiac Shunt	
Blalock-Taussig	52 (27%)
Sano	37 (19%)
Other	6 (3%)
N/A	97 (51%)
Residual Cardiac Lesions	
Yes	56 (29%)
No	136 (71%)
Returned to CICU on ECMO	
Yes	3 (2%)
Cardiac Arrest in CICU	14 (7%)
Chest Opened Postoperatively in CICU	31 (16%)
Arrhythmias	
Preoperative	12 (6%)
Intraoperative	29 (15%)
Postoperative	62 (32%)
Postoperative ECMO	8 (4%)
Duration of ECMO	0 [0–0]
Duration of Operative Intubation (hours)	32 [12–58]
Number of Intubations	1 [1–2]
Reintubation	37 (19%)
Delayed Sternal Closure	33 (17%)
Chest Re-Opened Postoperatively	19 (10%)
Return to OR for Chest Exploration	12 (6%)
Exploration with Cardiopulmonary Bypass	3 (25%)
Postoperative Chest Tubes	26 (14%)
Number of CICU Admissions	1 [1–1]
Total Length of CICU Stay	7 [5–12.5]
Total Length of Hospital Stay (days)	17 [12–26]
Diaphragm Paresis	4 (2%)
Postoperative Seizures	21 (11%)
Electroclinical Seizure	7 (33%)
Electrographic Seizure	14 (67%)
Chest Wound Infection	23 (12%)
Vocal Cord Paralysis	19 (10%)
Left	18 (95%)
Right	1 (5%)
Dialysis	0 (0%)

**Table 2.** Statistically significant variables in univariate analysis.

Variable	Entire Cohort p value (n = 192)	Group 1 p value (n = 188)	Group 2 p value (n = 188)	Group 4 p value (n = 188)
Primary CHD Grade	**<0.001**	——	——	——
Surgical Grade	**<0.001**	0.5610	0.4260	——
Normal Great Arteries	**<0.001**	0.0718	0.9400	0.0909
Transposed Great Arteries	**<0.001**	0.0718	0.9400	0.0909
Ascending Aortic Diameter (mm)	**<0.001**	0.4500	0.1170	0.6430
Was Cooling Performed?	**<0.001**	0.5750	——	——
Was Circulatory Arrest performed?	**<0.001**	0.5750	——	——
Total Support Time (Bypass þ DHCA)	**0.0013**	0.9860	0.2430	0.0001
Total Duration of DHCA (Circ Arrest)	**<0.001**	0.9460	0.1180	0.0016
Number of Times of DHCA	**<0.001**	0.9460	0.6940	0.5870
Calculated Total Duration of Bypass	0.2340	0.7220	0.2610	**0.0001**
Duration of Cooling (minutes)	**<0.001**	0.5610	0.2300	**0.0004**
Duration of Rewarming (minutes)	**<0.001**	0.5750	0.4640	**0.0003**
Lowest Temperature	**<0.001**	0.9810	0.3040	0.1020
Open Chest in CICU?	**0.0017**	0.1230	0.4460	0.0978
Type of Shunt	**0.0001**	0.1880	0.1790	0.9490
Duration of Initial Operative Intubation [Hours]	**<0.001**	0.1170	0.0525	**<0.001**
Delayed Sternal Closure (Did child come back from OR with chest open?)	**0.0027**	0.3050	0.5450	0.1340
Length of Chest Open	**0.0017**	0.3240	0.4560	0.0917
Total Length of CICU Stay (Days)	**<0.001**	0.0589	0.0138	0.0040
Length of Hospital Stay	**<0.001**	**0.0029**	0.0469	**0.0003**

(——): p value not calculated due to absence of variable within cohort.
