# Supplementary material for: Predictors of the inability to achieve full oral feeding in postoperative infants with CHD
Source: Cardiol Young. Author manuscript; Available in PMC 2026 Apr 22. (PMC13101751; doi:10.1017/S104795112300313X)
Supplement: Supplementary 1 [file NIHMS2153045-supplement-Supplementary_1.docx]

**Supplementary Table 1. Univariate Analysis**

| **Variable** | **Entire Cohort**  **p value**  (n=192) | **Group 1 p value**  (n=188) | **Group 2 p value**  (n=188) | **Group 4 p value**  (n=188) |
| --- | --- | --- | --- | --- |
| Gender | 0.1150 | 0.0423 | 0.1300 | 0.6620 |
| Race | 0.0126 | 0.7390 | 0.4030 | 0.0349 |
| Ethnicity | 0.6420 | 0.2280 | 0.4260 | 0.7380 |
| Head Circumference | 0.8660 | 0.2570 | 0.1460 | 0.2800 |
| Head Circumference z-score | 0.7180 | 0.4620 | 0.0337 | 0.4630 |
| Head Circumference Percentile | 0.7170 | 0.4650 | 0.0337 | 0.4560 |
| (weeks) | 0.7290 | 0.3290 | 0.5060 | 0.0690 |
| (days) | 0.2180 | 0.4920 | 0.8690 | 0.5280 |
| Birth Weight (kg) | 0.0186 | 0.7440 | 0.0317 | 0.1170 |
| Weight z-score | 0.0085 | 0.9350 | 0.0096 | 0.2880 |
| Weight Percentile | 0.0088 | 0.9440 | 0.0096 | 0.2960 |
| Length (cm) | 0.0286 | 0.9490 | 0.0058 | 0.9480 |
| Type of Birth | 0.8450 | 0.9030 | 0.8890 | 0.8580 |
| Reason For c-section | 0.6870 | 0.8010 | 0.9330 | 0.9410 |
| Pregnancy Complications: Gestational Diabetes | 0.5480 | 0.3050 | 0.2590 | 0.2490 |
| Pregnancy Complications: Gestational Hypertension | 0.3210 | 0.6670 | 0.5350 | 0.2490 |
| Pregnancy Complications: Pre-eclampsia | 0.2340 | ---- | 0.6940 | 0.5870 |
| Pregnancy Complications: Other | 0.6690 | 0.2370 | 0.2760 | 0.1170 |
| Pregnancy Complications: None | 0.7370 | 0.4510 | 0.6600 | 0.3710 |
| Pregnancy Complications Other: Tobacco | 0.9160 | 0.3500 | 0.5350 | 0.6210 |
| Pregnancy Complications Other: Hypothyroid | 0.1050 | 0.1750 | ---- | 0.7380 |
| Pregnancy Complications Other: Other | 0.4980 | 0.5750 | 0.4260 | 0.2370 |
| Fetal echo? | 0.5480 | 0.7990 | 0.3430 | 0.1930 |
| Gestational age at time of echo? | 0.2250 | 0.5100 | 0.3880 | 0.2850 |
| Uterine artery PI | 0.2920 | 0.9770 | 0.1100 | 0.8580 |
| Umbilical artery PI | 0.1540 | 0.3420 | 0.0559 | 0.6670 |
| Middle cerebral artery (MCA) PI | 0.5510 | 0.6490 | 0.1250 | 0.6800 |
| Placental pathology done? | 0.5470 | 0.9420 | 0.2760 | 0.5830 |
| Placental weight (g) | 0.8190 | 0.9110 | 0.1450 | 0.9730 |
| Placental infarcts? | 0.4970 | 0.5810 | 0.3750 | 0.6460 |
| Time of Diagnosis | 0.0473 | 0.9090 | 0.5350 | 0.5870 |
| Primary Diagnosis | 0.2580 | 0.0653 | 0.1740 | 0.4000 |
| Primary CHD Grade | **<0.001** | ---- | ---- | ---- |
| Surgical Grade | **<0.001** | 0.5610 | 0.4260 | ---- |
| Situs | 0.0400 | 0.5210 | ---- | ---- |
| Normal Great Arteries | **<0.001** | 0.0718 | 0.9400 | 0.0909 |
| Transposed Great Arteries | **<0.001** | 0.0718 | 0.9400 | 0.0909 |
| Great Artery Relations: AP Window | 0.4040 | 0.1440 | ---- | ---- |
| Great Artery Relations: Truncus | ---- | ---- | ---- | ---- |
| Right Ventricular Outflow Tract Obstruction | ---- | ---- | ---- | ---- |
| Left Ventricular Outflow Tract Obstruction | ---- | ---- | ---- | ---- |
| Left Distal Outflow Obstruction | ---- | ---- | ---- | ---- |
| Outflow Obstruction: Aberrant Branching | ---- | ---- | ---- | ---- |
| Outflow Obstruction: Right Side Aorta | ---- | ---- | ---- | ---- |
| Ascending Aortic Diameter (mm) | **<0.001** | 0.4500 | 0.1170 | 0.6430 |
| Normal Coronary Artery Anatomy | 0.1830 | 0.2800 | 0.3430 | 0.9860 |
| Anomalous Left Coronary Artery from the Pulmonary Artery (ALCAPA) | ---- | ---- | ---- | ---- |
| Single Coronary Artery | 0.9510 | 0.6670 | 0.6940 | 0.5870 |
| Aberrant Left Coronary Artery | 0.8160 | 0.5750 | ---- | ---- |
| Other Coronary Artery Abnormality | 0.4360 | 0.3940 | 0.3430 | 0.8390 |
| Pulmonary venous Return | 0.5020 | ---- | ---- | 0.9880 |
| Cardiac Catheter Intervention?(Preoperative Only) | 0.1630 | 0.0401 | 0.6940 | 0.8130 |
| Balloon or Stent | 0.2040 | 0.0401 | 0.6940 | 0.8230 |
| Preoperative oral feeding | 0.0149 | 0.3200 | 0.2216 | 0.0471 |
| Normal Behavior | 0.9430 | 0.4120 | ---- | 0.5870 |
| Abnormal Behavior | 0.3810 | 0.3500 | ---- | 0.5870 |
| Chromosomal Disorder | 0.0513 | 0.0173 | 0.6310 | 0.8520 |
| Specify Disorder | 0.0266 | 0.1940 | 0.1440 | ---- |
| Age at Surgery | 0.6300 | 0.3540 | 0.0324 | 0.0982 |
| Residual Cardiac Lesions Postop | 0.0324 | 0.6390 | 0.8890 | 0.0137 |
| Anesthesia Record: Was Cooling Performed? | **<0.001** | 0.5750 | ---- | ---- |
| Anesthesia Record: Was Circulatory Arrest performed? | **<0.001** | 0.5750 | ---- | ---- |
| Total Support Time (Bypass + DHCA) | **0.0013** | 0.9860 | 0.2430 | 0.0001 |
| Total Duration of DHCA (Circ Arrest) | **<0.001** | 0.9460 | 0.1180 | 0.0016 |
| Number of times of DHCA | **<0.001** | 0.9460 | 0.6940 | 0.5870 |
| Calculated Total Duration of Bypass | 0.2340 | 0.7220 | 0.2610 | **0.0001** |
| Number of Times on Bypass | 0.0897 | 0.0508 | 0.5350 | 0.1180 |
| Number of cross clamps | 0.7370 | 0.4120 | ---- | ---- |
| Calculated Cross Clamp time | 0.6660 | 0.8800 | 0.5250 | 0.0469 |
| Duration of Cooling (minutes) | **<0.001** | 0.5610 | 0.2300 | **0.0004** |
| Duration of Rewarming (minutes) | **<0.001** | 0.5750 | 0.4640 | **0.0003** |
| Calculated MUF time (minutes) | 0.8020 | 0.4050 | 0.4830 | 0.5410 |
| Lowest Temperature | **<0.001** | 0.9810 | 0.3040 | 0.1020 |
| Cardiac Arrest in CICU? | 0.4120 | 0.2450 | 0.2590 | 0.0920 |
| Open chest in CICU? | **0.0017** | 0.1230 | 0.4460 | 0.0978 |
| ECMO | 0.0580 | 0.5750 | 0.6940 | 0.1510 |
| Type of shunt | **0.0001** | 0.1880 | 0.1790 | 0.9490 |
| Preoperative Arrhythmia | 0.2290 | 0.9120 | 0.0411 | 0.4230 |
| Intraoperative Arrhythmia | 0.6600 | 0.8850 | 0.3110 | 0.8900 |
| Postoperative Arrhythmia | 0.0145 | 0.1260 | 0.2870 | 0.1870 |
| No Arrhythmia | 0.2460 | 0.6000 | 0.4350 | 0.4590 |
| Number of intubations | 0.2880 | 0.5270 | 0.0651 | 0.0703 |
| Duration of initial operative intubation [Hours, postoperative] | **<0.001** | 0.1170 | 0.0525 | **<0.001** |
| Delayed Sternal Closure (Did child come back from OR with chest open?) | **0.0027** | 0.3050 | 0.5450 | 0.1340 |
| Length of chest open | **0.0017** | 0.3240 | 0.4560 | 0.0917 |
| Chest re-opened postoperatively? | 0.1510 | 0.4900 | 0.2760 | 0.5070 |
| Length of Chest Open After Re-Opening | 0.1940 | 0.4480 | 0.2780 | 0.6130 |
| Cardiac Arrest Postoperative | 0.3050 | 0.2450 | 0.2590 | 0.0718 |
| Return to OR for Re-exploration? | 0.0709 | 0.5750 | 0.6940 | 0.3240 |
| ECMO Postop | 0.0889 | 0.5750 | 0.6940 | 0.1930 |
| Total time on ECMO | 0.0869 | 0.5610 | 0.6940 | 0.1940 |
| Post-Operative Chest Tubes? | 0.0039 | 0.1230 | 0.2220 | 0.1420 |
| Number of CICU admissions | 0.6420 | 0.3500 | 0.6940 | 0.8390 |
| Total Length of CICU Stay (days) | **<0.001** | 0.0589 | 0.0138 | 0.0040 |
| Length of Hospital Stay | **<0.001** | **0.0029** | 0.0469 | **0.0003** |
| Diaphragm Paresis | 0.0880 | ---- | 0.5350 | 0.4230 |
| Clinical Seizure | 0.4780 | 0.5210 | 0.6940 | 0.8130 |
| Subclinical Seizure | 0.3050 | 0.5750 | 0.4260 | 0.5330 |
| No Seizure | 0.1140 | 0.5750 | 0.3430 | 0.8390 |
| Seizure: Other | ---- | ---- | ---- | ---- |
| Chest Wound Infection | 0.2460 | 0.1710 | 0.4260 | 0.7220 |
| Vocal Cord Paralysis | 0.0039 | 0.0358 | 0.2220 | 0.5990 |
| Vocal Cord Paralysis: Left or Right | 0.0038 | 0.0358 | 0.2220 | 0.5860 |
| Dialysis | ---- | ---- | ---- | ---- |
| Type of MRI | 0.8140 | 0.5030 | ---- | 0.5800 |
| Total TMS Score | 0.9420 | 0.2900 | 0.6710 | 0.3370 |
| Total PVL Volume | 0.6850 | 0.9030 | 0.8560 | 0.9940 |
| ONLY if PVL volumetry not feasible: No PVL | ---- | ---- | ---- | ---- |
| ONLY if PVL volumetry not feasible: Right frontal PVL | ---- | ---- | ---- | ---- |
| ONLY if PVL volumetry not feasible: Left Frontal PVL | ---- | ---- | ---- | ---- |
| ONLY if PVL volumetry not feasible: Right Parietal PVL | ---- | ---- | ---- | ---- |
| ONLY if PVL volumetry not feasible: Left Parietal PVL location | ---- | ---- | ---- | ---- |
| Total QPS Score | 0.5980 | 0.9370 | 0.9420 | 0.9300 |
| Partial Right Posterior Cerebral Artery | ---- | ---- | ---- | ---- |
| Partial Left Posterior Cerebral Artery | ---- | ---- | ---- | ---- |
| Full Territory Right Posterior Cerebral Artery | ---- | ---- | ---- | ---- |
| Full Territory Left Posterior Cerebral Artery | ---- | ---- | ---- | ---- |
| Partial Right Middle Cerebral Artery | ---- | ---- | ---- | ---- |
| Partial Left Middle Cerebral Artery | 0.3780 | 0.5030 | ---- | 0.4310 |
| Full Territory Right Middle Cerebral Artery | ---- | ---- | ---- | ---- |
| Full Territory Left Middle Cerebral Artery | ---- | ---- | ---- | ---- |
| Partial Right Anterior Cerebral Artery | ---- | ---- | ---- | ---- |
| Partial Left Anterior Cerebral Artery | ---- | ---- | ---- | ---- |
| Full Territory Right Anterior Cerebral Artery | ---- | ---- | ---- | ---- |
| Full Territory Left Anterior Cerebral Artery | ---- | ---- | ---- | ---- |
| Tentorial/Posterior Fossa Subdural Hemorrhage | 0.5030 | 0.8140 | 0.2270 | 0.4020 |
| Right Frontal Subdural Hemorrhage | ---- | ---- | ---- | ---- |
| Left Frontal Subdural Hemorrhage | ---- | ---- | ---- | ---- |
| Right Parietal Subdural Hemorrhage | ---- | ---- | ---- | ---- |
| Left Parietal Subdural Hemorrhage | 0.4060 | ---- | ---- | 0.5800 |
| Right Temporal Subdural Hemorrhage | ---- | ---- | ---- | ---- |
| Left Temporal Subdural Hemorrhage | ---- | ---- | ---- | ---- |
| Right Occipital Subdural Hemorrhage | 0.2010 | 0.4850 | 0.4070 | 0.5630 |
| Left Occipital Subdural Hemorrhage | 0.1740 | 0.4380 | 0.3220 | 0.9670 |
| Interhemispheric Subdural Hemorrhage | 0.2210 | 0.5380 | 0.2910 | 0.5800 |
| Tentorial/Posterior Fossa Subarachnoid Hemorrhage | ---- | ---- | ---- | ---- |
| Right Frontal Subarachnoid Hemorrhage | 0.4060 | ---- | ---- | 0.5800 |
| Left Frontal Subarachnoid Hemorrhage | 0.4060 | 0.1620 | ---- | ---- |
| Right Parietal Subarachnoid Hemorrhage | ---- | ---- | ---- | ---- |
| Left Parietal Subarachnoid Hemorrhage | ---- | ---- | ---- | ---- |
| Right Temporal Subarachnoid Hemorrhage | 0.2400 | ---- | ---- | 0.0913 |
| Left Temporal Subarachnoid Hemorrhage | ---- | ---- | ---- | ---- |
| Right Occipital Subarachnoid Hemorrhage | ---- | ---- | ---- | ---- |
| Left Occipital Subarachnoid Hemorrhage | ---- | ---- | ---- | ---- |
| Interhemispheric Subarachnoid Hemorrhage | 0.4060 | ---- | ---- | 0.5800 |
| Left Choroid Plexus | 0.5120 | 0.8140 | 0.3880 | 0.2400 |
| Right Choroid Plexus | 0.3060 | 0.9720 | 0.3050 | 0.1880 |
| Left Germinal Matrix | 0.2350 | ---- | 0.6830 | 0.5800 |
| Right Germinal Matrix | 0.6730 | ---- | 0.5180 | 0.0206 |
| Germinal Matrix Hemorrhage Grade I | 0.8170 | ---- | 0.4070 | 0.0691 |
| Germinal Matrix Hemorrhage Grade II | ---- | ---- | ---- | ---- |
| Germinal Matrix Hemorrhage Grade III | ---- | ---- | ---- | ---- |
| Germinal Matrix Hemorrhage Grade IV | ---- | ---- | ---- | ---- |
| Left Frontal Parenchymal Hemorrhage | ---- | ---- | ---- | ---- |
| Right Frontal Parenchymal Hemorrhage | ---- | ---- | ---- | ---- |
| Left Temporal Parenchymal Hemorrhage | 0.2400 | ---- | ---- | ---- |
| Right Temporal Parenchymal Hemorrhage | 0.4060 | ---- | ---- | 0.5800 |
| Left Parietal Parenchymal Hemorrhage | ---- | ---- | ---- | ---- |
| Right Parietal Parenchymal Hemorrhage | ---- | ---- | ---- | ---- |
| Left Occipital Parenchymal Hemorrhage | ---- | ---- | ---- | ---- |
| Right Occipital Parenchymal Hemorrhage | 0.2400 | ---- | ---- | ---- |
| Left Cerebellar Parenchymal Hemorrhage | 0.4060 | ---- | 0.6830 | ---- |
| Right Cerebellar Parenchymal Hemorrhage | 0.4060 | 0.1620 | ---- | ---- |
| Subcortical Parenchymal Hemorrhage | ---- | ---- | ---- | ---- |
| Left Frontal Parenchymal Microhemorrhage | 0.7880 | 0.4650 | ---- | 0.2720 |
| Right Frontal Parenchymal Microhemorrhage | 0.3960 | 0.3300 | ---- | 0.4460 |
| Left Temporal Parenchymal Microhemorrhage | 0.5020 | ---- | 0.0500 | 0.3150 |
| Right Temporal Parenchymal Microhemorrhage | 0.5020 | 0.5030 | 0.6830 | 0.4190 |
| Left Parietal Parenchymal Microhemorrhage | 0.4060 | ---- | ---- | 0.5800 |
| Right Parietal Parenchymal Microhemorrhage | 0.3780 | 0.5030 | ---- | 0.4310 |
| Left Occipital Parenchymal Microhemorrhage | 0.2350 | ---- | ---- | 0.4190 |
| Right Occipital Parenchymal Microhemorrhage | 0.7770 | ---- | 0.6830 | 0.5800 |
| Total number of Microhemorrhages | 0.8170 | 0.6190 | 0.7850 | 0.2660 |
| Right Transverse Sinus Venous Thrombosis | ---- | ---- | ---- | ---- |
| Left Transverse Sinus Venous Thrombosis | ---- | ---- | ---- | ---- |
| Sagittal Sinus Venous Thrombosis | ---- | ---- | ---- | ---- |
| Brain MRI summary findings | 0.7260 | 0.5000 | 0.7960 | 0.5500 |
| Total brain volume (cTBV) without CSF, calculated (mm3, mcl) | 0.1670 | 0.3170 | 0.0261 | 0.2770 |
| Brainstem (mm3,mcl) | 0.4320 | 0.6440 | 0.0065 | 0.8300 |
| Cerebellum (mm3,mcl) | 0.2940 | 0.4610 | 0.0519 | 0.4890 |
| Gray Matter/ Cortex (mm3,mcl) | 0.3210 | 0.5160 | 0.2230 | 0.1950 |
| Deep Gray Matter (mm3, mcl) | 0.4360 | 0.4350 | 0.0183 | 0.6050 |
| White Matter (mm3, mcl) | 0.3330 | 0.1340 | 0.0103 | 0.5420 |
| Infratentorial CSF (mm3, mcl) | 0.4120 | 0.5820 | 0.0314 | 0.3560 |
| Ventricular CSF (mm3, mcl) | 0.5340 | 0.8510 | 0.0737 | 0.3620 |
| Supratentorial CSF (mm3, mcl) | 0.5730 | 0.9300 | 0.0314 | 0.3680 |
| Volume Caudate Nucleus Left (mm3/mcl) | 0.9680 | 0.6320 | 0.1700 | 0.3010 |
| Volume Caudate Nucleus Right (mm3/mcl) | 0.9740 | 0.6320 | 0.1700 | 0.4320 |
| Age at Postop MRI (days) | 0.0190 | 0.0946 | 0.1950 | 0.8820 |
| Type of MRI | 0.0344 | 0.2120 | 0.2880 | 0.6130 |
| Total TMS Score | 0.2860 | 0.4760 | ---- | ---- |
| Total PVL Volume | 0.0907 | 0.4950 | 0.9730 | 0.3290 |
| ONLY if PVL volumetry not feasible: No PVL | 0.1420 | ---- | 0.7600 | 0.4060 |
| ONLY if PVL volumetry not feasible: Right Frontal | 0.4040 | ---- | ---- | 0.5710 |
| ONLY if PVL volumetry not feasible: Left Frontal PVL | 0.2420 | 0.5050 | ---- | ---- |
| ONLY if PVL volumetry not feasible: Right Parietal PVL | 0.4040 | ---- | ---- | 0.5710 |
| ONLY if PVL volumetry not feasible: Left Parietal PVL | 0.4040 | ---- | ---- | 0.5710 |
| Total QPS Score | 0.0764 | 0.4560 | 0.8650 | 0.4340 |
| Partial Right Posterior Cerebral Artery | 0.4040 | ---- | ---- | 0.5710 |
| Partial Left Posterior Cerebral Artery | ---- | ---- | ---- | ---- |
| Full Territory Right Posterior Cerebral Artery | ---- | ---- | ---- | ---- |
| Full Territory Left Posterior Cerebral Artery | ---- | ---- | ---- | ---- |
| Partial Right Middle Cerebral Artery | 0.1760 | 0.5050 | ---- | 0.1130 |
| Partial Left Middle Cerebral Artery | 0.7410 | 0.5050 | ---- | 0.7960 |
| Full Territory Right Middle Cerebral Artery | ---- | ---- | ---- | ---- |
| Full Territory Left Middle Cerebral Artery | ---- | ---- | ---- | ---- |
| Partial Right Anterior Cerebral Artery | 0.4040 | ---- | ---- | 0.5710 |
| Partial Left Anterior Cerebral Artery | 0.4040 | ---- | ---- | 0.5710 |
| Full Territory Right Anterior Cerebral Artery | ---- | ---- | ---- | ---- |
| Full Territory Left Anterior Cerebral Artery | ---- | ---- | ---- | ---- |
| Tentorial/Posterior Fossa Subdural Hemorrhage | 0.5670 | 0.0946 | 0.2390 | 0.5650 |
| Right Frontal Subdural Hemorrhage | 0.2420 | ---- | ---- | 0.1010 |
| Left Frontal Subdural Hemorrhage | ---- | ---- | ---- | ---- |
| Right Parietal Subdural Hemorrhage | ---- | ---- | ---- | ---- |
| Left Parietal Subdural Hemorrhage | 0.4040 | ---- | ---- | 0.5710 |
| Right Temporal Subdural Hemorrhage | ---- | ---- | ---- | ---- |
| Left Temporal Subdural Hemorrhage | 0.4040 | 0.1620 | ---- | ---- |
| Right Occipital Subdural Hemorrhage | 0.5810 | 0.3780 | 0.4170 | 0.6020 |
| Left Occipital Subdural Hemorrhage | 0.6740 | 0.5120 | 0.3470 | 0.4390 |
| Interhemispheric Subdural Hemorrhage | 0.0153 | 0.0213 | 0.5010 | 0.2270 |
| Tentorial/Posterior Fossa Subarachnoid Hemorrhage | 0.4040 | ---- | ---- | 0.5710 |
| Right Frontal Subarachnoid Hemorrhage | 0.4040 | 0.1620 | ---- | ---- |
| Left Frontal Subarachnoid Hemorrhage | 0.4040 | ---- | 0.7600 | ---- |
| Right Parietal Subarachnoid Hemorrhage | ---- | ---- | ---- | ---- |
| Left Parietal Subarachnoid Hemorrhage | ---- | ---- | ---- | ---- |
| Right Temporal Subarachnoid Hemorrhage | 0.2420 | ---- | ---- | 0.1010 |
| Left Temporal Subarachnoid Hemorrhage | ---- | ---- | ---- | ---- |
| Right Occipital Subarachnoid Hemorrhage | ---- | ---- | ---- | ---- |
| Left Occipital Subarachnoid Hemorrhage | ---- | ---- | ---- | ---- |
| Interhemispheric Subarachnoid Hemorrhage | ---- | ---- | ---- | ---- |
| Left Choroid Plexus | 0.1820 | 0.7910 | 0.6640 | 0.4550 |
| Right Choroid Plexus | 0.1820 | 0.7910 | 0.6640 | 0.4550 |
| Left Germinal Matrix | 0.4040 | ---- | 0.7600 | ---- |
| Right Germinal Matrix | 0.4060 | ---- | 0.7600 | 0.0252 |
| Germinal Matrix Hemorrhage Grade I | 0.6820 | ---- | 0.6070 | 0.0252 |
| Germinal Matrix Hemorrhage Grade II | ---- | ---- | ---- | ---- |
| Germinal Matrix Hemorrhage Grade III | ---- | ---- | ---- | ---- |
| Germinal Matrix Hemorrhage Grade IV | ---- | ---- | ---- | ---- |
| Left Frontal Parenchymal Hemorrhage | ---- | ---- | ---- | ---- |
| Right Frontal Parenchymal Hemorrhage | 0.4040 | ---- | 0.7600 | ---- |
| Left Temporal Parenchymal Hemorrhage | 0.2420 | ---- | ---- | ---- |
| Right Temporal Parenchymal Hemorrhage | 0.4040 | ---- | ---- | 0.5710 |
| Left Parietal Parenchymal Hemorrhage | ---- | ---- | ---- | ---- |
| Right Parietal Parenchymal Hemorrhage | 0.4040 | ---- | ---- | 0.5710 |
| Left Occipital Parenchymal Hemorrhage | ---- | ---- | ---- | ---- |
| Right Occipital Parenchymal Hemorrhage | 0.2420 | ---- | ---- | ---- |
| Left Cerebellar Parenchymal Hemorrhage | 0.8190 | 0.5050 | 0.7600 | ---- |
| Right Cerebellar Parenchymal Hemorrhage | 0.4040 | 0.1620 | ---- | ---- |
| (----): p value not calculated due to absence of variable within cohort | | | | |
