# Supplementary material for: Predictors of the inability to achieve full oral feeding in postoperative infants with CHD
Source: Cardiol Young. Author manuscript; Available in PMC 2026 Apr 22. (PMC13101751; doi:10.1017/S104795112300313X)
Supplement: Supplementary 2 [file NIHMS2153045-supplement-Supplementary_2.docx]

Supplementary Table 2. Independent Variables

**Prenatal Variables**

| Variable | Variable Coding | Primary Study | Secondary Analysis | Level of Measurement | Level of Analysis |
| --- | --- | --- | --- | --- | --- |
| Date of Admission | Y-M-D | X | X | Interval | Continuous |
| Date of Birth | Y-M-D | X | X | Interval | Continuous |
| Time of Birth | H:M | X | X | Interval | Continuous |
| Date of Consent | Y-M-D | X | X | Interval | Continuous |
| Gender | 0, Female  1, Male | X | X | Nominal | Dichotomous |
| Race | 1, African American/Caribbean/African  2, Asian  3, Caucasian  4, Native American/Pacific Islander  5, Mixed  6, Other  7, Unknown | X | X | Nominal | Dichotomous |
| Ethnicity | 1, Latino  2, Other | X | X | Nominal | Dichotomous |
| Head Circumference | Centimeters | X | X | Ratio | Continuous |
| Head Circumference z-score |  | X | X | Ratio | Continuous |
| Head Circumference Percentile |  | X | X | Ratio | Continuous |
| Gestational Age | Weeks/days | X | X | Ratio | Continuous |
| Birth Weight | Kilograms | X | X | Ratio | Continuous |
| Weight z-score |  | X | X |  |  |
| Weight Percentile |  | X | X | Ratio | Continuous |
| Length | Centimeters | X | X | Ratio | Continuous |
| Type of Birth | 1, Vaginal  2, C-section | X | X | Nominal | Dichotomous |
| Reason for c-section | 1, Failure to progress  2, Repeat elective  3, Breech  4, Emergency  5, Other | X | X | Nominal | Categorical |
| Pregnancy Complications | 1, Gestational Diabetes  2, Gestational Hypertension  3, Pre-eclampsia  4, Other  5, None | X | X | Nominal | Categorical |
| Pregnancy Complications Other Categories | 1, Tobacco  2, Hypothyroid  3, Other | X | X | Nominal | Categorical |
| Fetal echo? | Yes/no | X | X | Nominal | Dichotomous |
| Date of fetal echo | Y-M-D | X |  |  |  |
| Gestational age at time of echo | Weeks/days | X | X | Ratio | Continuous |
| Uterine Artery Pulsatility Index | Free text number | X | X | Ratio | Continuous |
| Umbilical Artery Pulsatility Index | Free text number | X | X | Ratio | Continuous |
| Middle Cerebral Artery Pulsatility Index | Free text number | X | X | Ratio | Continuous |
| Placental Pathology done? | Yes/no | X | X | Nominal | Dichotomous |
| Placental weight | Grams | X | X | Ratio | Continuous |
| Placental infarcts? | Yes/no | X | X | Nominal | Dichotomous |
| Time of cardiac diagnosis | 1, Prenatal  2, Postnatal | X | X | Nominal | Dichotomous |
| Primary cardiac diagnosis | 1, HLHS  2, d-TGA  3, IAA  4, Truncus  5, TOF/PA  6, Coarctation/VSD  7, Ebstein’s  8, Unbalanced AV canal  9, DILV  10, DORV  11, Aortic arch hypoplasia/VSD  12, Aortic arch hypoplasia/coarctation  13, TOF (without PA)  14, Pulmonary atresia  15, Tricuspid atresia | X | X | Nominal | Dichotomous |
| Secondary cardiac diagnosis | 1, HLHS  2, d-TGA  3, IAA  4, Truncus  5, TOF/PA  6, Coarctation/VSD  7, Ebstein’s  8, Unbalanced AV canal  9, DILV  10, DORV  11, Aortic arch hypoplasia/VSD  12, Aortic arch hypoplasia/coarctation  13, TOF (without PA)  14, Pulmonary atresia  15, Tricuspid atresia | X |  | Nominal | Dichotomous |
| Primary CHD Grade | 1, I (2 ventricles, normal arch)  2, II (2 ventricles, arch obstruction)  3, III (1 ventricle, normal arch)  4, IV (1 ventricle, arch obstruction) | X | X | Nominal | Dichotomous |
| Surgical Grade | 1, I (Corrective, no DHCA)  2, II (Corrective, DHCA)  3, III (Palliative, no DHCA)  4, IV (Palliative, DHCA) | X | X | Nominal | Dichotomous |
| Situs | 1, Solitus  2, Inversus | X | X | Nominal | Dichotomous |
| Great artery relations | 1, Normal  2, Transposed  3, AP Window  4, Truncus | X | X | Nominal | Dichotomous |
| Outflow obstruction | 2, RVOTO  3, LVOTO  4, Left distal obstruction  5, Aberrant branching  6, Right side aorta | X | X | Nominal | Dichotomous |
| RVOTO type | 1, Stenosis  2, Atresia | X | X | Nominal | Dichotomous |
| LVOTO type | 1, Stenosis  2, Atresia | X | X | Nominal | Dichotomous |
| Left distal obstruction type | 1, Pre-ductal  2, post-ductal | X | X | Nominal | Dichotomous |
| Ascending aortic diameter | Millimeters | X | X | Ratio | Continuous |
| Coronary artery anatomy | 1, Normal  2, Abnormal | X | X | Nominal | Dichotomous |
| Coronary artery abnormalities | 1, ALCAPA  2, Single coronary  3, Aberrant left  4, Other | X | X | Nominal | Dichotomous |
| Pulmonary venous return | 1, Normal  2, Total anomalous  3, Partial anomalous | X | X | Nominal | Dichotomous |

**Preoperative Procedures**

| Variable | Variable Coding | Primary Study | Secondary Analysis | Level of Measurement | Level of Analysis |
| --- | --- | --- | --- | --- | --- |
| Pre-surgical procedures | 1, Intubation prior to day of surgery  2, Extubation prior to surgery  3, Balloon Atrial Septostomy  4, Cardiac catheterization  5, Other (specify)  6, None | X |  | Nominal | Continuous |
| Intubation for reason other than procedure? | Yes/no | X | X | Nominal | Dichotomous |
| Reason for intubation? | Free text note | X | X |  |  |
| Intubation (non-procedural) start date/time | Y-M-D H:M | X |  |  |  |
| Extubation (non-procedural) date/time | Y-M-D H:M | X |  |  |  |
| Length of Non-procedural intubation | Calculated field | X | X | Ratio | Continuous |
| BAS Date | Y-M-D | X |  |  |  |
| BAS Location | 1, Bedside  2, Interventional Suite  3, Outside Hospital | X |  | Nominal | Dichotomous |
| Other pre-surgical procedures | Free text note | X |  |  |  |
| Cardiac Catheter intervention (pre-operative only) | Yes/no | X | X | Nominal | Dichotomous |
| Balloon or Stent | 1, Balloon  2, Stent  3, Other | X | X | Nominal | Dichotomous |
| Cardiac catheterization date/time | Y-M-D H:M | X |  | Interval | Continuous |

**Preoperative Clinical Exam**

| Variable | Variable Coding | Primary Study | Secondary Analysis | Level of Measurement | Level of Analysis |
| --- | --- | --- | --- | --- | --- |
| Behavior | 1, Normal  2, Abnormal | X | X | Nominal | Dichotomous |
| Abnormal Behavior Comments | Free text note | X | X |  |  |
| Chromosomal Disorder | 1, Yes  2, No  3, Suspected | X | X | Nominal | Dichotomous |
| Specify Disorder | Free text note | X | X |  |  |
| Extra-Cardiac Anomalies | 1, Skin  2, Eyes  3, Ears  4, Mouth/Jaw  5, Arms  6, Legs  7, Genitals  8, Anus  9, Lungs  10, Liver  11, Kidney  12, Intestine  13, Spine  14, None | X |  | Nominal | Dichotomous |
| Skin Abnormalities | 1, Port-of-Wine Stain  2, Hemangioma  3, Nevus  4, Other | X |  | Nominal | Dichotomous |
| Skin Abnormality Comment | Free text note | X |  |  |  |
| Hemangioma Type | 1, Segmental (V-1)  2, Segmental (V-2)  3, Segmental (V-3)  4, Mouth  5, Neck  6, Body | X |  | Nominal | Dichotomous |
| Nevus Location | Free text note | X |  |  |  |
| Eye Abnormalities | Free text note | X |  |  |  |
| Ear Abnormalities | Free text note | X |  |  |  |
| Mouth/Jaw Abnormalities | Free text note | X |  |  |  |
| Arm Abnormalities | Free text note | X |  |  |  |
| Leg Abnormalities | Free text note | X |  |  |  |
| Genital Abnormalities | Free text note | X |  |  |  |
| Anus Abnormalities | Free text note | X |  |  |  |
| Lung Abnormalities | Free text note | X |  |  |  |
| Liver Abnormalities | Free text note | X |  |  |  |
| Kidney Abnormalities | Free text note | X |  |  |  |
| Intestine Abnormalities | Free text note | X |  |  |  |
| Spine Abnormalities | Free text note | X |  |  |  |

**Brain MRIs (Preoperative/Postoperative)**

| Variable | Variable Coding | Primary Study | Secondary Analysis | Level of Measurement | Level of Analysis |
| --- | --- | --- | --- | --- | --- |
| Received MRI | Yes/no | X | X | Nominal | Dichotomous |
| Type of MRI | 0, Pre/Op MRI clinical  1, Pre-Op MRI research  2, Post-Op MRI research  3, Post-Op MRI clinical  4, other imaging | X | X | Nominal | Dichotomous |
| Reason no MRI | 1, ECMO therapy  2, seizures  3, stroke/hemorrhage on pre-MRI  4, multi organ failure  5, drop out /parents declined MRI  6, no MRI due to medical instability  7, scheduling/administrative issue  8, scan attempted, but aborted due to motion artifacts/intolerance, not rescheduled  99, unknown | X |  | Nominal | Dichotomous |
| Quality of MRI for TMS/ PVL volumes | 1, usable  2, not usable or not accurate for TMS/PVL volumes | X |  | Nominal | Dichotomous |
| Additional information on the scan quality | 1, motion degraded  2, clinical scan  3, other | X | X | Nominal | Dichotomous |
| MRI Quality Notes | Free text note | X | X |  |  |
| MRI Date | Y-M-D | X |  | Interval | Continuous |
| MRI Time | H:M | X |  | Interval | Continuous |
| SWI grading | 0, not feasible  1, mild  2, moderate  3, severe | X | X | Nominal | Dichotomous |
| Total Germinal Matrix Score | Free text number | X | X | Ratio | Continuous |
| Total Cortical Folding Score | Free text number | X | X | Ratio | Continuous |
| Total Myelination Score | Free text number | X | X | Ratio | Continuous |
| Total Migrating Glia Score | Free text number | X | X | Ratio | Continuous |
| Total TMS Score | Calculated field | X | X |  | Continuous |
| Left Frontal (mm^3^volume) | Free text number | X |  | Ratio | Continuous |
| Left Parietal (mm^3^volume) | Free text number | X |  | Ratio | Continuous |
| Right Frontal (mm^3^volume) | Free text number | X |  | Ratio | Continuous |
| Right Parietal (mm^3^volume) | Free text number | X |  | Ratio | Continuous |
| Total PVL Volume | Calculated field | X | X | Ratio | Continuous |
| ONLY if PVL volumetry not feasible: PVL location | 0, no PVL  1, Right frontal  2, Left frontal  3, Right parietal  4, Left parietal | X | X | Nominal | Dichotomous |
| QPS score (check ALL that apply) | 0, no PVL  1, Is PVL present?  2, Is there PVL in all 4 quadrants?  3, Are there 3 or more lesions in 1 quadrant?  4, Are any of the lesions greater than 5mm (axial view)? | X | X | Nominal | Dichotomous |
| Total QPS Score | Calculated field | X | X | Ratio | Continuous |
| Wallerian Degeneration | 1, Anterior Corpus Callosum  2, Splenium  3, Thalamus (Left)  4, Thalamus (Right)  5, Corticospinal Tract (Left)  6, Corticospinal Tract (Right) | X |  | Nominal | Dichotomous |
| Brain Stem | 1, Medulla  2, Pons  3, Midbrain | X |  | Nominal | Dichotomous |
| Cerebellar | 1, PICA (Right)  2, PICA (Left)  3, AICA (Right)  4, AICA (Left)  5, SCA (Right)  6, SCA (Left) | X |  | Nominal | Dichotomous |
| Subcortical Brain Matter | 1, Thalamus (Right)  2, Thalamus (Left)  3, Basal Ganglia (Right)  4, Basal Ganglia (Left)  5, Caudate (Right)  6, Caudate (Left) | X |  | Nominal | Dichotomous |
| Posterior Cerebral Artery | 1, Partial (Right)  2, Partial (Left)  3, Full Territory (Right)  4, Full Territory (Left) | X | X | Nominal | Dichotomous |
| Media Cerebral Artery | 1, Partial (Right)  2, Partial (Left)  3, Full Territory (Right)  4, Full Territory (Left) | X | X | Nominal | Dichotomous |
| Anterior Cerebral Artery | 1, Partial (Right)  2, Partial (Left)  3, Full Territory (Right)  4, Full Territory (Left) | X | X | Nominal | Dichotomous |
| Subdural Hemorrhage | 1, Tentorial / Posterior Fossa  2, Frontal (Right)  3, Frontal (Left)  4, Parietal (Right)  5, Parietal (Left)  6, Temporal (Right)  7, Temporal (Left)  8, Occipital (Right)  9, Occipital (Left)  10, Inter-hemispheric | X | X | Nominal | Dichotomous |
| Subarachnoid Hemorrhage | 1, Tentorial / Posterior Fossa  2, Frontal (Right)  3, Frontal (Left)  4, Parietal (Right)  5, Parietal (Left)  6, Temporal (Right)  7, Temporal (Left)  8, Occipital (Right)  9, Occipital (Left)  10, Inter- hemispheric | X | X | Nominal | Dichotomous |
| Choroid Plexus | 1, Left  2, Right | X | X | Nominal | Dichotomous |
| Germinal Matrix | 1, Left  2, Right | X | X | Nominal | Dichotomous |
| Germinal Matrix Hemorrhage Grade | 1, I confined to the germinal matrix  2, II intraventricular hemorrhage without ventricular dilatatation  3, III intraventricular hemorrhage with ventricular dilatation  4, IV GMH with intraventricular rupture and hemorrhage into the surrounding white matter | X | X | Nominal | Dichotomous |
| Parenchymal Hemorrhage | 1, Frontal (Left)  2, Frontal (Right)  3, Temporal (Left)  4, Temporal (Right)  5, Parietal (Left)  6, Parietal (Right)  7, Occipital (Left)  8, Occipital (Right)  9, Cerebellar (Left)  10, Cerebellar (Right)  11, Subcortical Areas | X | X | Nominal | Dichotomous |
| Parenchymal Microhemorrhage | 1, Frontal (Left)  2, Frontal (Right)  3, Temporal (Left)  4, Temporal (Right)  5, Parietal (Left)  6, Parietal (Right)  7, Occipital (Left)  8, Occipital (Right) | X | X | Nominal | Dichotomous |
| Central Structures Microhemorrhage | 1, Thalamus (Left)  2, Thalamus (Right)  3, Hippocampus (Left)  4, Hippocampus (Right)  5, Basal Ganglia (Left)  6, Basal Ganglia (Right)  7, Midbrain (Left)  8, Midbrain (Right) | X |  | Nominal | Dichotomous |
| Cerebellum Microhemorrhage | 1, Right  2, Left | X |  | Nominal | Dichotomous |
| Brain Stem Microhemorrhage | 1, Right  2, Left | X |  | Nominal | Dichotomous |
| Total number of Microhemorrhages | Free text number | X | X | Ratio | Continuous |
| Sinus Venous Thrombosis | 1, Transverse (Right)  2, Transverse (Left)  3, Sagittal | X | X | Nominal | Dichotomous |
| Clinical MRI Exam Notes from Neuroradiologist | Free text note | X |  |  |  |
| Brain MRI summary findings | 0, Unremarkable  1, PVL/WMI ONLY  2, PVL/WMI + other abnormal finding  3, Other abnormal finding ONLY (PVL/WMI not present)  4, PVL/WMI present BUT conventional analysis not feasible  5, no brain MRI done | X |  | Nominal | Dichotomous |
| Brain mask volume (brain volume and CSF) (mm3,mcl) | Free text number | X |  | Ratio | Continuous |
| Total intracranial volume (cTIV), calculated (mm3,mcl) | Calculated field | X |  | Ratio | Continuous |
| Total brain volume (cTBV) without CSF, calculated (mm3, mcl) | Calculated field | X | X | Ratio | Continuous |
| Brainstem (mm3,mcl) | Free text number | X | X | Ratio | Continuous |
| Cerebellum (mm3,mcl) | Free text number | X | X | Ratio | Continuous |
| Gray Matter/ Cortex (mm3,mcl) | Free text number | X | X | Ratio | Continuous |
| Deep Gray Matter (mm3, mcl) | Free text number | X | X | Ratio | Continuous |
| White Matter (mm3, mcl) | Free text number | X | X | Ratio | Continuous |
| Infratentorial CSF (mm3, mcl) | Free text number | X |  | Ratio | Continuous |
| Ventricular CSF (mm3, mcl) | Free text number | X |  | Ratio | Continuous |
| Supratentorial CSF (mm3, mcl) | Free text number | X |  | Ratio | Continuous |
| Volume Caudate Nucleus Left (mm3/mcl) | Free text number | X | X | Ratio | Continuous |
| Volume Caudate Nucleus Right (mm3/mcl) | Free text number | X | X | Ratio | Continuous |
| FA Caudate Nucleus Left | Free text number | X |  | Ratio | Continuous |
| FA Caudate Nucleus Right | Free text number | X |  | Ratio | Continuous |
| MD Caudate Nucleus Left | Free text number | X |  | Ratio | Continuous |
| MD Caudate Nucleus Right | Free text number | X |  | Ratio | Continuous |
| AD Caudate Nucleus Left | Free text number | X |  | Ratio | Continuous |
| AD Caudate Nucleus Right | Free text number | X |  | Ratio | Continuous |
| RD Caudate Nucleus Left | Free text number | X |  | Ratio | Continuous |
| RD Caudate Nucleus Right | Free text number | X |  | Ratio | Continuous |
| FA MEAN Cerebellum | Free text number | X |  | Ratio | Continuous |
| SD FA Cerebellum | Free text number | X |  | Ratio | Continuous |
| RD Cerebellum | Free text number | X |  | Ratio | Continuous |
| FA MEAN Whole brain | Free text number | X |  | Ratio | Continuous |
| FA SD Whole brain | Free text number | X |  | Ratio | Continuous |
| RD Whole brain | Free text number | X |  | Ratio | Continuous |
| Reason no analysis | 1, not feasible due to structural anomalies  2, no T2w/T1w scan for volumetry  10, no DTI sequence  11, software failure  12, DTI signal direction failure  13, DTI signal distorted | X |  | Nominal | Dichotomous |

**Intraoperative Variables**

| Variable | Variable Coding | Primary Study | Secondary Analysis | Level of Measurement | Level of Analysis |
| --- | --- | --- | --- | --- | --- |
| Date of Surgery | Y-M-D | X |  | Ratio | Continuous |
| Age at Surgery | Calculated field | X | X | Ratio | Continuous |
| Surgeon | 1, Spray  2, Gaynor  3, Fuller  4, Gruber  6, Mascio  5, Other | X |  | Nominal | Dichotomous |
| Other Surgeon | Free text | X |  |  |  |
| Residual Cardiac Lesions | Yes/no | X | X | Nominal | Dichotomous |
| Anesthesia Start Time (induction) | H:M | X |  | Ratio | Continuous |
| Anesthesia Record: start total support time(bypass cannulation time) | H:M | X |  | Ratio | Continuous |
| Anesthesia Record: Was Cooling Performed? | 0, no  1, yes | X | X | Nominal | Dichotomous |
| Anesthesia Record: start of cooling | H:M | X |  | Ratio | Continuous |
| Stop of Cooling | H:M | X |  | Ratio | Continuous |
| Anesthesia Record: Was Circulatory Arrest performed? | 0, no  1, yes | X | X | Nominal | Dichotomous |
| Cross Clamp on Time | H:M | X |  | Ratio | Continuous |
| Anesthesia Record: start Circ Arrest (same as stop of cooling) | H:M | X |  | Ratio | Continuous |
| Anesthesia Record: stop Circ Arrest (same as start of warming) | H:M | X |  | Ratio | Continuous |
| Cross Clamp Off Time | H:M | X |  | Ratio | Continuous |
| Anesthesia Record: stop of warming | H:M | X |  | Ratio | Continuous |
| Anesthesia Record: stop total support time (final bypass stop time) | H:M | X |  | Ratio | Continuous |
| MUF start (indicated on bypass record) | H:M | X |  | Ratio | Continuous |
| MUF stop (may show up as either MUF stop, or MUF volume on bypass record) | H:M | X |  | Ratio | Continuous |
| Total Support Time (Bypass + DHCA) | Calculated field | X | X | Ratio | Continuous |
| Calculated Duration of Total Length of DHCA (Circ Arrest) | Calculated field | X | X | Ratio | Continuous |
| Calculated Duration of Bypass | Calculated field | X | X | Ratio | Continuous |
| Calculated Cross Clamp time | Calculated field | X | X | Ratio | Continuous |
| Duration of Cooling (minutes) | Calculated field | X | X | Ratio | Continuous |
| Duration of Rewarming (minutes) | Calculated field | X | X | Ratio | Continuous |
| Calculated MUF time (minutes) | Calculated field | X | X | Ratio | Continuous |
| Additional support time?(back on bypass) | 0, no  1, yes | X | X | Nominal | Dichotomous |
| Anesthesia Record: Time on Bypass Run 2 | H:M | X |  | Ratio | Continuous |
| Anesthesia Record: Time off Bypass Run 2 | H:M | X |  | Ratio | Continuous |
| Calculated Total Length of Bypass 2 (minutes) | Calculated field | X | X | Ratio | Continuous |
| Calculated Cross Clamp Time Run 2 | Calculated field | X | X | Ratio | Continuous |
| Anesthesia Record: Time on Bypass Run 3 | H:M | X |  | Ratio | Continuous |
| Anesthesia Record: Time off Bypass Run 3 | H:M | X |  | Ratio | Continuous |
| Calculated Total Length of Bypass 3 (minutes) | Calculated field | X | X | Ratio | Continuous |
| Calculated Cross Clamp Time Run 3 | Calculated field | X | X | Ratio | Continuous |
| Additional DHCA? | 0, no  1, yes | X | X | Nominal | Dichotomous |
| Additional Cross Clamp Start Time 2 | H:M | X |  | Ratio | Continuous |
| Anesthesia Record: Time on Circ Arrest 2 | H:M | X |  | Ratio | Continuous |
| Additional Cross Clamp Time off 2 | H:M | X |  | Ratio | Continuous |
| Anesthesia Record: Time off Circ Arrest 2 | H:M | X |  | Ratio | Continuous |
| Additional Cross Clamp Start Time 3 | H:M | X |  | Ratio | Continuous |
| Anesthesia Record: Time on Circ Arrest 3 | H:M | X |  | Ratio | Continuous |
| Additional Cross Clamp Time off 3 | H:M | X |  | Ratio | Continuous |
| Anesthesia Record: Time off Circ Arrest 3 | H:M | X |  | Ratio | Continuous |
| Calculated Total DHCA 2 | Calculated field | X | X | Ratio | Continuous |
| Calculated Total DHCA 3 | Calculated field | X | X | Ratio | Continuous |
| Lowest Temperature | Celsius | X | X | Ratio | Continuous |
| Cardiac Arrest in CICU? | Yes/no | X | X | Nominal | Dichotomous |
| Open chest in CICU? | Yes/no | X | X | Nominal | Dichotomous |
| ECMO | Yes/no | X | X | Nominal | Dichotomous |
| ECMO in CICU? | Yes/no | X | X | Nominal | Dichotomous |
| On ECMO returning from surgery? | Yes/no | X | X | Nominal | Dichotomous |
| Cannula placement | 1, Neck  2, Chest | X | X | Nominal | Dichotomous |
| ECLS mode | 1, VV  2, VA | X | X | Nominal | Dichotomous |
| Type of shunt | 1, BT  2, Sano  3, Other  4, N/A | X | X | Nominal | Dichotomous |
| Peri-Op Arrhythmia | 1, Preoperative  2, Intraoperative  3, Postoperative\|4, None | X | X | Nominal | Dichotomous |
| Preoperative Arrhythmia Comment | Free text note | X | X |  |  |
| Intraoperative Arrhythmia Comment | Free text note | X | X |  |  |
| Postoperative Arrhythmia Comment | Free text note | X | X |  |  |
| Ventilator Mode | 1, Volume Control  2, Pressure Control | X |  | Nominal | Dichotomous |
| Respiratory Rate |  | X |  | Ratio | Continuous |
| PEEP (mmHg) |  | X |  | Ratio | Continuous |
| FiO2 (ratio, 0.21 - 1.00) |  | X |  | Ratio | Continuous |
| EtCO2 (mmHg) |  | X |  | Ratio | Continuous |
| PIP (mmHg) |  | X |  | Ratio | Continuous |
| Tidal Volume (ml) |  | X |  | Ratio | Continuous |
| Minute Ventilation |  | X |  | Ratio | Continuous |
| Timing of Blood Gas | 0, unknown  1, birth  2, before cath intervention/Balloon Atrial septostomy (BAS)  3, before surgery  4, during surgery  5, post-surgery | X |  | Nominal | Dichotomous |
| BGA during general anesthesia | Yes/no | X |  | Nominal | Dichotomous |
| Inspired Oxygen (%, range 21-100%) | Free text number | X |  | Ration | Continuous |
| Type of Blood Gas | 1, ABG  2, VBG  3, Cap Gas | X |  | Nominal | Dichotomous |
| Blood Gas date/time | Y-M-D, H:M | X |  | Ratio | Continuous |
| pH | Free text number | X |  | Ratio | Continuous |
| pCO2 (mmHg) | Free text number | X |  | Ratio | Continuous |
| pO2 (mmHg) | Free text number | X |  | Ratio | Continuous |
| HCO3- (mmol/L) | Free text number | X |  | Ratio | Continuous |
| BE (mmol/L) | Free text number | X |  | Ratio | Continuous |
| SaO2 (ABG est.) | Free text number | X |  | Ratio | Continuous |
| Hct |  | X |  | Ratio | Continuous |
| Hemoglobin (g/dL) | Free text number | X |  | Ratio | Continuous |
| Hemoglobin (iStat) | Free text number | X |  | Ratio | Continuous |
| Na (mmol/L) | Free text number | X |  | Ratio | Continuous |
| K (mmol/L) | Free text number | X |  | Ratio | Continuous |
| Ca (mmol/L) | Free text number | X |  | Ratio | Continuous |
| SaO2 (Co-ox) (%) | Free text number | X |  | Ratio | Continuous |
| Notes | Free text | X |  |  |  |
| Was preoperative CBC done? | 0, No  1, Yes | X |  | Nominal | Dichotomous |
| What timeframe was preoperative CBC done relative to surgery? | 1, Within 24 hours of surgery  2, Within 48 hours of surgery  3, Within 72 hours of surgery | X |  | Nominal | Dichotomous |
| Preoperative CBC date/time |  | X |  | Nominal | Dichotomous |
| WBC |  | X |  | Ratio | Continuous |
| Hemoglobin |  | X |  | Ratio | Continuous |
| Hematocrit |  | X |  | Ratio | Continuous |
| Platelet Count |  | X |  | Ratio | Continuous |
| Postoperative CBC (CBC closest to surgery) | 1, Within 24 hours after surgery  2, Within 48 hours after surgery  3, Within 72 hours after surgery | X |  | Nominal | Dichotomous |
| WBC |  | X |  | Ratio | Continuous |
| Hemoglobin |  | X |  | Ratio | Continuous |
| Hematocrit |  | X |  | Ratio | Continuous |
| Platelet Count |  | X |  | Ratio | Continuous |

**Postoperative Variables**

| Variable | Variable Coding | Primary Study | Secondary Analysis | Level of Measurement | Level of Analysis |
| --- | --- | --- | --- | --- | --- |
| Initial Operative Intubation Date | Y-M-D | X |  | Ratio | Continuous |
| First Postoperative Extubation Date/Time | H:M | X |  | Ratio | Continuous |
| Duration of initial operative intubation [Hours, post-operative] | Calculated Field | X | X | Ratio | Continuous |
| Reintubation | Yes/no | X | XX | Nominal | Dichotomous |
| Reason for Reintubation? | 1, Cardiac  2, Respiratory  3, Seizures  4, Other | X |  | Nominal | Dichotomous |
| If other, Reintubation reason? | Free text note | X | X |  |  |
| Reintubation1 Time | H:M | X |  | Ratio | Continuous |
| Reintubation1 Extubation Time | H:M | X |  | Ratio | Continuous |
| Duration of post-operative reintubation [Hours] | Calculated field | X | X | Ratio | Continuous |
| Additional second post-operative Reintubation | Yes/no | X | X | Nominal | Dichotomous |
| Reason for second post-operative Reintubation? | 1, Respiratory  2, Cardiac  3, Seizures  4, Other | X | X | Nominal | Dichotomous |
| If other, Reintubation reason? | Free text note | X | X |  |  |
| Reintubation2 Time | H:M | X |  | Ratio | Continuous |
| Reintubation2 Extubation Time | H:M | X |  | Ratio | Continuous |
| Duration of second post-operative intubation [Hours] | Calculated field | X | X | Ratio | Continuous |
| Additional Reintubation | Yes/no | X | X | Nominal | Dichotomous |
| Reason for Reintubation? | 1, Respiratory  2, Cardiac  3, Seizures  4, Other | X | X | Nominal | Dichotomous |
| If other, Reintubation reason? | Free text note | X | X |  |  |
| Reintubation3 Time | H:M | X |  | Ratio | Continuous |
| Reintubation3 Extubation Time | H:M | X |  | Ratio | Continuous |
| Duration of third post-operative intubation [Hours] | Calculated field | X | X | Ratio | Continuous |
| Additional Intubations? | Yes/no | X | X | Nominal | Dichotomous |
| Reason for additional Reintubations? | 1, Respiratory  2, Cardiac  3, Seizures  4, Other | X | X | Nominal | Dichotomous |
| Additional Reintubation Notes | Free text note | X | X |  |  |
| Delayed Sternal Closure(Did child come back from OR with chest open?) | Yes/no | X | X | Nominal | Dichotomous |
| Chest Closure | Y-M-D H:M | X |  | Ratio | Continuous |
| Length of Chest Open | Calculated field |  | X | Ratio | Continuous |
| Chest re-opened post-operatively? | Yes/no | X | X | Nominal | Dichotomous |
| Chest Re-opening Date/Time | Y-M-D H:M | X |  | Ratio | Continuous |
| Chest Closure after Re-Opening | Y-M-D H:M | X |  | Ratio | Continuous |
| Length of Chest Open After Re-Opening | Calculated field |  | X | Ratio | Continuous |
| Chest Closure Note | Free text note | X | X |  |  |
| Cardiac Arrest Post-Op | Yes/no | X | X | Nominal | Dichotomous |
| Cardiac Arrest Postop Note | Free text note | X | X |  |  |
| Return to OR for Re-exploration? | Yes/no | X | X | Nominal | Dichotomous |
| Re-Exploration with Bypass? | Yes/no | X | X | Nominal | Dichotomous |
| Date and Time of re-exploration Date/Time | Y-M-D H:M | X |  | Ratio | Continuous |
| Surgical Re-Exploration Note | Free text note | X | X |  |  |
| ECMO Postop | Yes/no | X | X | Nominal | Dichotomous |
| Postop ECMO Start Date and Time | Y-M-D H:M | X |  | Ratio |  |
| Postop ECMO Stop Date/Time | Y-M-D H:M | X |  | Ratio |  |
| Total Time on ECMO | Calculated field |  | X | Ratio | Continuous |
| ECMO Note | Free text note | X | X |  |  |
| Post-Operative Chest Tubes? | Yes/no | X | X | Nominal | Dichotomous |
| Post-Operative Chest Tube Insertion | Y-M-D H:M | X |  | Ratio | Continuous |
| Post-Operative Chest Tube Removal | Y-M-D H:M | X |  | Ratio | Continuous |
| Additional Post-Operative Chest Tubes? | Yes/no | X | X | Nominal | Dichotomous |
| Post-Operative Chest Tube Notes | Free text note | X | X |  |  |
| Return from Surgery Date/Time | Y-M-D H:M | X |  | Ratio | Continuous |
| Discharge from CICU | Y-M-D H:M | X |  | Nominal | Dichotomous |
| Initial Post-Op CICU Length of Stay | Calculated field | X | X | Ratio | Continuous |
| Total Length of CICU Stay | Calculated field | X | X | Ratio | Continuous |
| Readmission to CICU? | Yes/no | X | X | Nominal | Dichotomous |
| Readmission to CICU Date/Time | Y-M-D H:M | X |  | Ratio | Continuous |
| Readmission to CICU Note | Free text note | X | X |  |  |
| Subsequent Discharge from CICU | Y-M-D H:M | X |  | Ratio | Continuous |
| Second Post-Op CICU Stay (Precise) | Calculated field | X | X | Ratio | Continuous |
| Discharge from Hospital (Date) | Y-M-D | X |  | Ratio | Continuous |
| Length of Stay | Calculated field | X | X | Ratio | Continuous |
| Deceased Date | Y-M-D | X |  | Ratio | Continuous |
| Diaphragm Paresis | Yes/no | X | X | Nominal | Dichotomous |
| Diaphragm Paresis Note | Free text note | X | X |  |  |
| Seizure | 1, Clinical Seizure  2, Subclinical Seizure  3, None  4, Other | X | X | Nominal | Dichotomous |
| Seizure Note | Free text note | X | X |  |  |
| Chest Wound Infection | Yes/no | X | X | Nominal | Dichotomous |
| Chest Wound Infection Note | Free text note | X | X |  |  |
| Vocal Cord Paralysis | Yes/no | X | X | Nominal | Dichotomous |
| Vocal Cord Paralysis: Left or Right | 1, Left  2, Right | X | X | Nominal | Dichotomous |
| Dialysis | Yes/no | X | X | Nominal | Dichotomous |
| Other Notable Events | Free text note | X | X |  |  |

**Central Venous/Arterial Lines (Preoperative/Intraoperative/Postoperative)**

| Variable | Variable Coding | Primary Study | Secondary Analysis | Level of Measurement | Level of Analysis |
| --- | --- | --- | --- | --- | --- |
| Type of Line | 1, UAC (Umbilical Arterial Catheter)  2, Arterial Line  3, RA (Right Atrial) Line 1  4, UVC (Umbilical Venous Catheter)  5, Other Central Line  6, RA (Right Atrial) Line 2  7, None | X |  | Nominal | Dichotomous |
| "Other" Central Line Type | 1, Femoral  2, IJ  3, PICC line | X |  | Nominal | Dichotomous |
| Date/Time of UAC Insertion | Y-M-D H:M | X |  | Ratio | Continuous |
| Date/Time of UAC Removal | Y-M-D H:M | X |  | Ratio | Continuous |
| Total UAC Line Time (hours) | Calculated field | X |  | Ratio | Continuous |
| Date/Time of Arterial Line (not UAC) Insertion | Y-M-D H:M | X |  | Ratio | Continuous |
| Date/Time of Arterial Line (not UAC) Removal | Y-M-D H:M | X |  | Ratio | Continuous |
| Total Arterial Line (not UAC) Time (hours) | Calculated field | X |  | Ratio | Continuous |
| Date/Time RA Line Insertion 1 | Y-M-D H:M | X |  | Ratio | Continuous |
| Date/Time of RA Line Removal 1 | Y-M-D H:M | X |  | Ratio | Continuous |
| Date/Time RA Line Insertion 2 | Y-M-D H:M | X |  | Ratio | Continuous |
| Date/Time RA Line Removal 2 | Y-M-D H:M | X |  | Ratio | Continuous |
| Total RA Line 2 Time (hours) | Calculated field | X |  | Ratio | Continuous |
| Total RA Line 1 Time (hours) | Calculated field | X |  | Ratio | Continuous |
| Date/Time of UVC Insertion | Y-M-D H:M | X |  | Ratio | Continuous |
| Date/Time of UVC Removal | Y-M-D H:M | X |  | Ratio | Continuous |
| Total UVC Line Time (hours) | Calculated field | X |  | Ratio | Continuous |
| Date/Time Femoral Line Insertion | Y-M-D H:M | X |  | Ratio | Continuous |
| Date/Time Femoral Line Removal | Y-M-D H:M | X |  | Ratio | Continuous |
| Total Femoral Line Time | Calculated field | X |  | Ratio | Continuous |
| Date/Time IJ Insertion | Y-M-D H:M | X |  | Ratio | Continuous |
| Date/Time of IJ Removal | Y-M-D H:M | X |  | Ratio | Continuous |
| Total IJ Line Time (hours) | Calculated field | X |  | Ratio | Continuous |
| Date/Time of PICC Line Insertion | Y-M-D H:M | X |  | Ratio | Continuous |
| Date/Time of PICC Line Removal | Y-M-D H:M | X |  | Ratio | Continuous |
| Total PICC Line Time (hours) | Calculated field | X |  | Ratio | Continuous |
| Notes on line (complications, etc.) | Free text note | X |  |  |  |
